# Supplementary material for: Augmented microglial endoplasmic reticulum-mitochondria contacts mediate depression-like behavior in mice induced by chronic social defeat stress
Source: Nat Commun. 2024 Jun 18;15:5199. doi: 10.1038/s41467-024-49597-z (PMC11189428; doi:10.1038/s41467-024-49597-z)
Supplement: Supplementary file 1 — Supplementary Information [file 41467_2024_49597_MOESM1_ESM.pdf]

Augmented microglial endoplasmic reticulum-mitochondria contacts mediate depression-like behavior in mice induced by chronic social defeat stress

Supplemental Information

Supplementary Figure 1-10

Supplementary Table 1

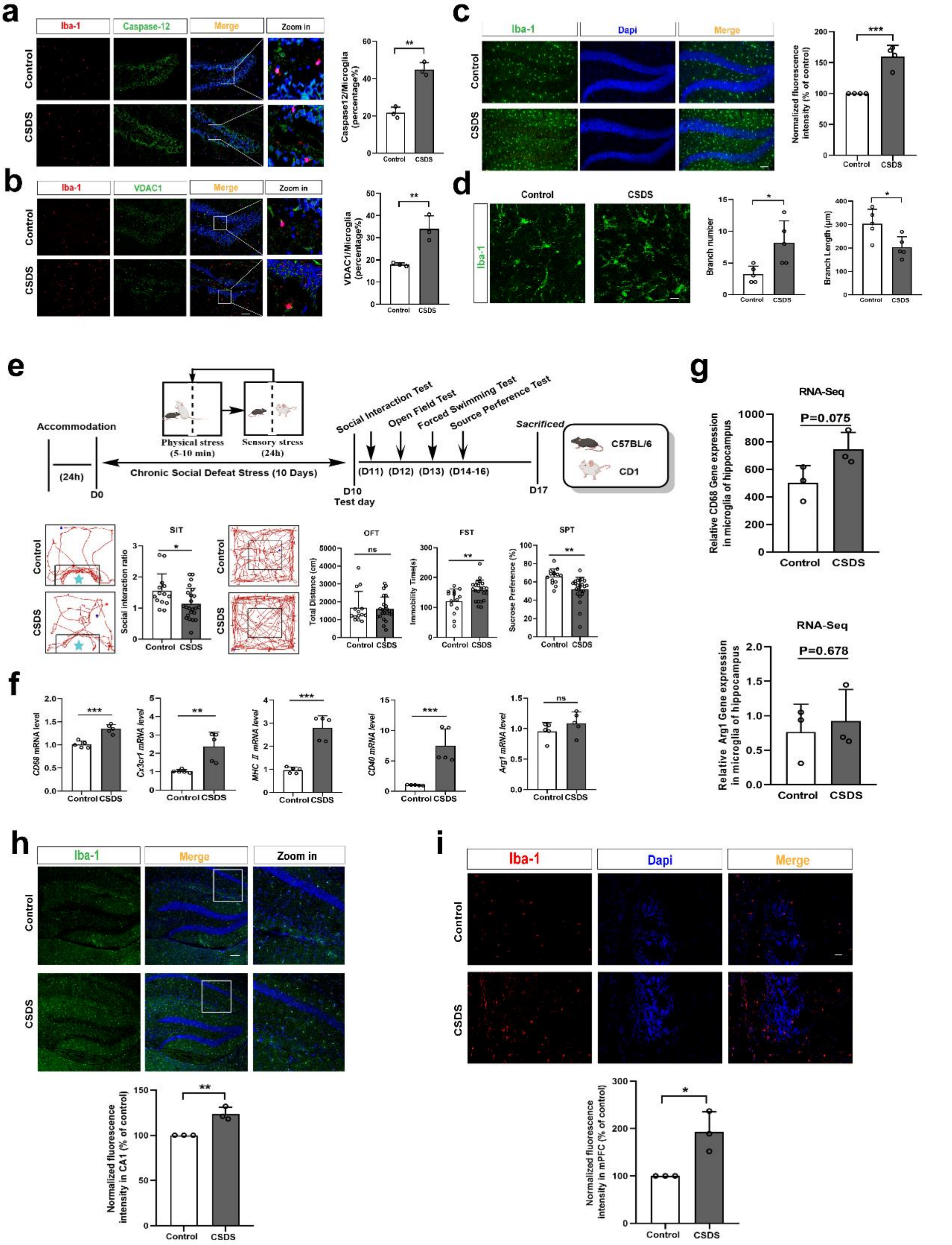

Supplementary Figure 1. Related to Figure 1: Chronic stress induces depressive-like behaviors in mice, microglial reaction, ER stress and mitochondrial damage in hippocampal microglia.

a. Representative immunofluorescent images (left panel) and percentage of positive

microglia (right panel) of Caspase-12 in Iba-1 cells in hippocampal DG from Control and CSDS mice. Caspase-12 (green), Iba-1 (red), DAPI (blue). Scale bar: 20 μm; Student's *t*-test (P = 0.0011). Data are expressed as mean ± SEM (n = 3 mouse brain slices). **, $p < 0.01$.

b. Representative immunofluorescent images (left panel) and percentage of positive microglia (right panel) of VDAC1 in Iba-1 cells in hippocampal DG from Control and CSDS mice. VDAC1 (green), Iba-1 (red), DAPI (blue). Scale bar: 20 μm; Student's *t*-test (P = 0.0096). Data are expressed as mean ± SEM (n = 3 mouse brain slices). **, $p < 0.01$.

c. Representative immunofluorescent images (left panel) and semi-quantitative analysis (right panel) of Iba-1 fluorescence intensity of hippocampal microglia in dentate gyrus (DG) from control and CSDS mice. Iba-1 (green), DAPI (blue). Scale bar: 20 μm; Student's *t*-test (P = 0.0005). Data are expressed as mean ± SEM (n = 4 mouse brain slices). ***$p < 0.001$.

d. Representative confocal fluorescence images of the morphology of microglia in the hippocampus of control and CSDS mice (left panel) and the statistics on the number and length of branches of microglia (right panel). Scale bar: 20 μm; Student's *t*-test (P = 0.0157; P = 0.0192). Data are expressed as mean ± SEM (n = 5 mouse brain slices). *$p < 0.05$.

e. Schematic (sFigure 1e was created with BioRender.com released under a Creative Commons Attribution-Non Commercial-No Derivs 4.0 International license) showing the 10-day chronic social defeat stress (CSDS) paradigm. Behavioral tests including

Social Interaction Test (SIT, P = 0.0248), open-field test (OFT, P = 0.7657), forced swim test (FST, P = 0.0051) and sucrose preference test (SPT, P = 0.0010) in Control (n = 20) and CSDS mice (n = 14 or 23) were performed. Student's *t*-test. Data are expressed as mean ± SEM. ns, $p > 0.05$, *$p < 0.05$, **$p < 0.01$.

f. Real-time PCR analysis of *CD68, CX3CR1*, *MHC II, CD40*, and *Arg1* in the control and CSDS group. Student's *t*-test (P = 0.00022; P = 0.0049; P < 0.0001; P = 0.0009; P = 0.2409). Data are expressed as mean ± SEM (n = 5). ns, $p > 0.05$, **$p < 0.01$, ***$p < 0.001$.

g. RNA-sequencing analysis of the expression levels of *CD68* and *Arg1* in microglia of the hippocampus in the control and CSDS group. Student's *t*-test. Data are expressed as mean ± SEM (n = 3). Ten hippocampi from 5 mice were combined to one sample.

h. Representative immunofluorescent images (left panel) and semi-quantitative analysis (right panel) of Iba-1 fluorescence intensity of hippocampal microglia in CA1 from control and CSDS mice. Iba-1 (green), DAPI (blue). Scale bar: 20 μm; Student's *t*-test (P = 0.0051). Data are expressed as mean ± SEM (n = 3 mouse brain slices). **$p < 0.01$.

i. Representative immunofluorescent images (left panel) and semi-quantitative analysis (right panel) of Iba-1 fluorescence intensity of hippocampal microglia in mPFC from control and CSDS mice. Iba-1 (red), DAPI (blue). Scale bar: 20 μm; Student's *t*-test (P = 0.0197). Data are expressed as mean ± SEM (n = 3 mouse brain slices). *$p < 0.05$. Source data are provided as a Source Data file.

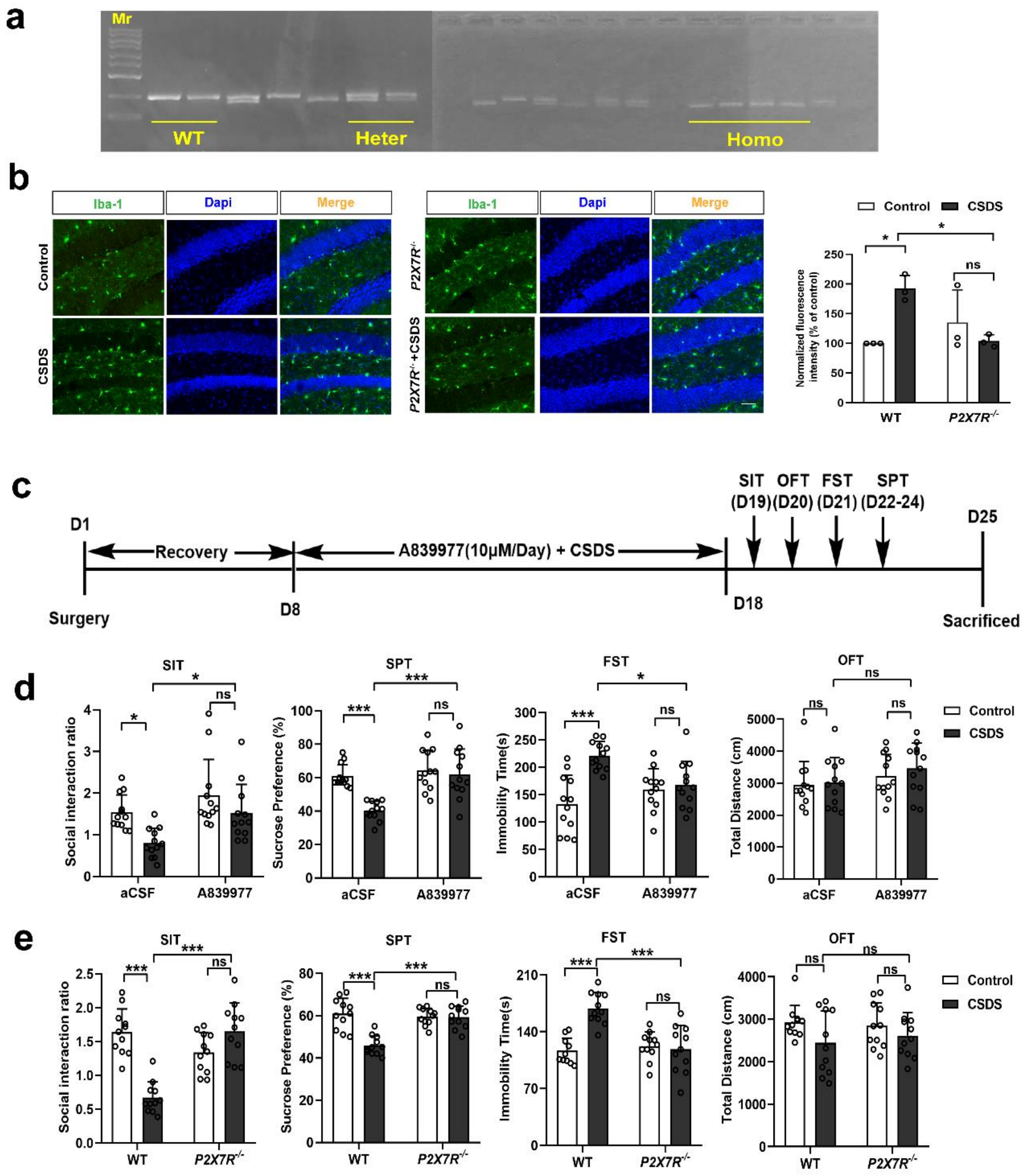

Supplementary Figure 2. Related to Figure 2 and 5: Purinergic receptor P2X7 (P2X7R) mediate microglia activation and depressive-like behavior.

a. Genotypes of the P2X7R heterozygote (Heter) and homozygote (Homo) mice were determined by PCR analysis.

b. Immunofluorescent analysis of Iba-1in the hippocampal DG of wildtype (WT) and *P2X7R*$^{-/-}$ mice with or without CSDS exposure were performed. Iba-1 (green), DAPI

(blue). Scale bar: 20 μm; Two-way ANOVA with Tukey's multiple comparisons test (Interaction, $F_{(1, 8)} = 12.54$, P = 0.0076). Data are expressed as mean ± SEM (n = 3 mouse brain slices). ns, $p > 0.05$, *$p < 0.05$.

c. Experimental timeline of surgery, recovery, CSDS, drug injection and behavioral testing.

d. Blockade of P2X7R with A839977 attenuates the development of the depressive-like behaviors induced by CSDS. Behavioral tests, including SIT, SPT, FST, and OFT were performed. Two-way ANOVA with Sidak's multiple comparisons test (Interaction, $F_{(1, 44)} = 0.7147$, P = 0.4025; Interaction, $F_{(1, 44)} = 9.019$, P = 0.0044; Interaction, $F_{(1, 44)} = 11.52$, P = 0.0015; Interaction, $F_{(1, 44)} = 0.1440$, P = 0.7061). Data are expressed as mean ± SEM (n = 3). *$p < 0.05$. (n = 12). ns, $p > 0.05$, *$p < 0.05$, ***$p < 0.001$.

e. Knockout of *P2X7R* results in increased resilience to CSDS. Behavioral tests in WT and $P2X7R^{-/-}$ mice with or without CSDS exposure were performed. Two-way ANOVA with Tukey's multiple comparisons test (Interaction, $F_{(1, 40)} = 41.84$, P < 0.0001; Interaction, $F_{(1, 40)} = 20.52$, P < 0.0001; Interaction, $F_{(1, 40)} = 19.41$, P < 0.0001; Interaction, $F_{(1, 40)} = 0.5171$, P = 0.4763). Data are expressed as mean ± SEM (n = 11). ns, $p > 0.05$, ***$p < 0.001$. Source data are provided as a Source Data file.

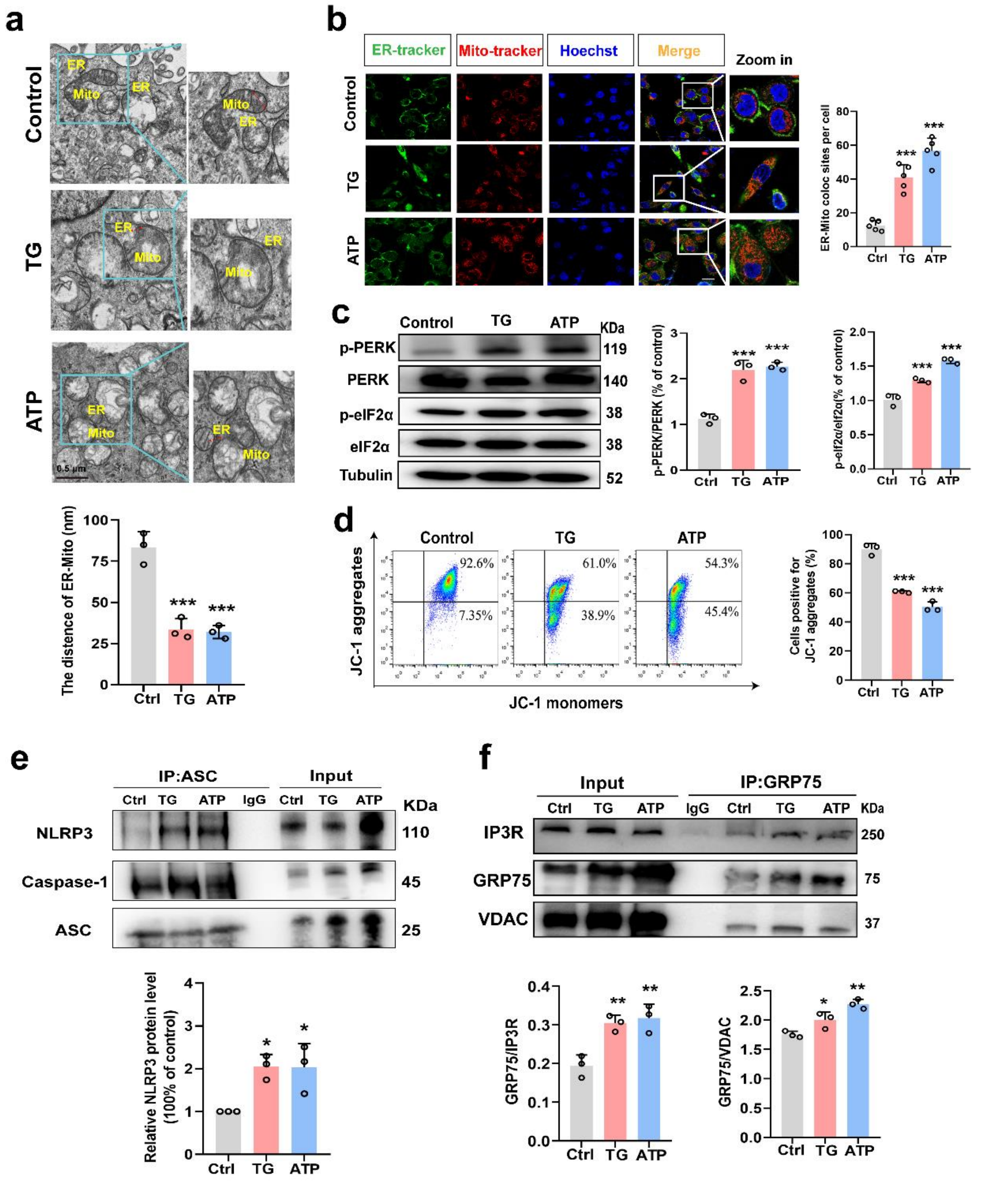

Supplementary Figure 3. Related to Figure 2: Extracellular ATP (eATP) leads to ER stress, alterations in MAMs, and mitochondria damage in BV2 cells.

a. Ultrastructural analysis of ER-mitochondria contact in BV2 cells. Representative Transmission Electronic Microscope (TEM) images of ER-mitochondria contact (above panel) and the quantitative analysis of the distance between mitochondria and the ER (below panel). Scale bar: 0.5 or 0.25 μm; one-way ANOVA with Dunnett's

multiple comparisons test (P = 0.0003; P = 0.0002). Data are expressed as mean ± SEM (n = 3 mitochondria in 3 fields per condition). ****p* < 0.001, *vs.* Ctrl.

b. Representative confocal images (left panel) and quantification of the colocalization between endoplasmic reticulum (ER) and mitochondria (right panel) of BV2 cells stained for nuclei (Hoechst 33342, blue), ER (ER-Tracker, green), and mitochondria (Mito-Tracker, red). Scale bar: 20 μm; One-way ANOVA with Dunnett's multiple comparisons test (P < 0.0001; P < 0.0001). Data are expressed as mean ± SEM (n = 3 different sets of experiments per condition). ****p* < 0.001, *vs.* Ctrl.

c. Representative western blot images (left panel) and statistical analysis (right panel) of PERK and elf2α phosphorylation in BV2 cells untreated (Ctrl) or treated with TG or ATP. One-way ANOVA with Dunnett's multiple comparisons test (P = 0.0002; P = 0.0002; P = 0.0016; P < 0.0001). Data are expressed as mean ± SEM (n = 3). ****p* < 0.001, *vs.* Ctrl.

d. Flow cytometric analysis of the relative protein level of mitochondrial membrane potential (MMP) in BV2 cells. Left panel: Representative flow cytometry plots of BV2 cells stained with JC-1 after treated with 1 μM TG or 1 mM ATP. Right panel: the percentage of JC-1 aggregate-positive cells in each group. One-way ANOVA with Dunnett's multiple comparisons test (P < 0.0001; P < 0.0001). Data are expressed as mean ± SEM (n = 3 per condition). ****p* < 0.001, *vs*. Ctrl.

e. Representative blots of co-IP (above panel) and quantification of the protein changes of NLRP3 and Caspase-1 in three groups, One-way ANOVA with Dunnett's multiple comparisons test (P = 0.0212; P = 0.0226). Data are expressed as mean ± SEM (n = 3).

*$p < 0.05$, *vs*. Ctrl.

f. Representative western blot analysis of total lysates and anti-GRP75 immunoprecipitants (IP) from BV2 cells with or without (Control) TG and ATP treatment respectively (above panel) and densitometric analysis of the ratio of co-immunoprecipitated GRP75/IP3R or GRP75/VDAC (right panel). one-way ANOVA with Dunnett's multiple comparisons test (P = 0.0061; P = 0.0034; P = 0.0369; P = 0.0011). Data are expressed as mean ± SEM (n = 3). *$p < 0.05$, **$p < 0.01$, *vs.* Ctrl. Source data are provided as a Source Data file.

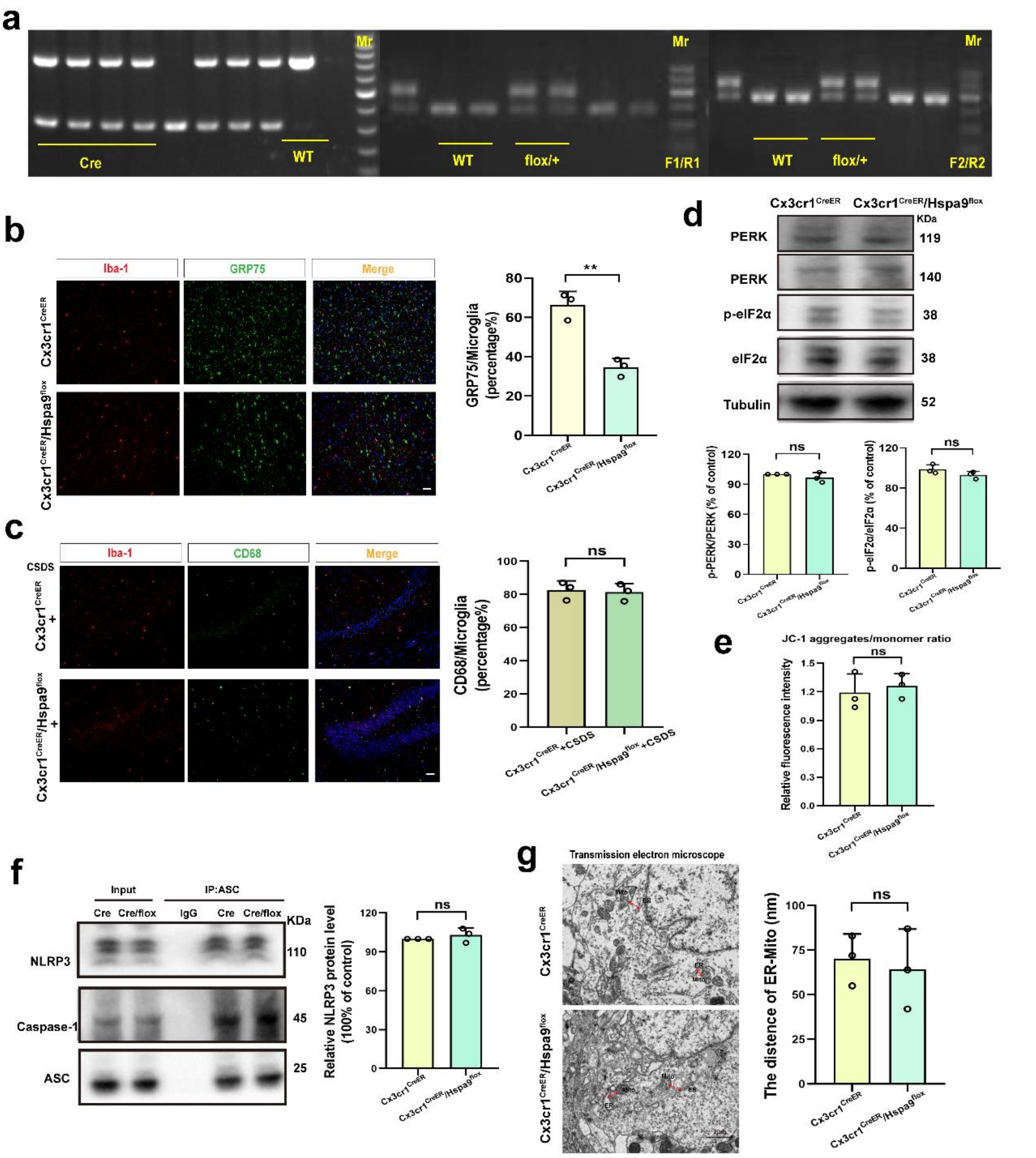

Supplementary Figure 4. Related to Figure 4: The cell-type specific knockout of GRP75 in mice.

a. Genotypes of the Cx3cr1$^{CreER/+}$ and Hspa9$^{f/+}$ mice were determined by PCR analysis.

b. Representative immunofluorescent images (left panel) and percentage of positive microglia (right panel) of GRP75 in Iba-1 cells in mPFC from Cx3cr1$^{CreER/+}$ and Cx3cr1$^{CreER/+}$/Hspa9$^{f/+}$ mice. GRP75 (green), Iba-1 (red), DAPI (blue). Scale bar: 20 µm; Student's *t*-test (P = 0.0307). Data are expressed as mean ± SEM (n = 3 mouse

brain slices). **, $p < 0.01$.

c. Representative immunofluorescent images (left panel) and percentage of positive microglia (right panel) of CD68 in Iba-1 cells in hippocampal DG from $Cx3cr1^{CreER/+}$ and $Cx3cr1^{CreER/+}/Hspa9^{f/+}$ mice treated with CSDS. CD68 (green), Iba-1 (red), DAPI (blue). Scale bar: 20 μm; Student's *t*-test (P = 0.7979). Data are expressed as mean ± SEM (n = 3 mouse brain slices). ns, $p > 0.05$.

d. Western blot analysis of PERK and elf2α phosphorylation proteins from $Cx3cr1^{CreER/+}$ mice or $Cx3cr1^{CreER/+}/Hspa9^{f/+}$ mice (n = 3 mouse tissue samples). Student's *t*-test (P = 0.3153; P = 0.1740). Data are expressed as mean ± SEM (n = 3 mouse brain slices). ns, $p > 0.05$.

e. Ratios of JC-1 aggregates to JC-1 monomer in two groups (n = 3 mouse tissue samples). Student's *t*-test (P = 0.6346). Data are expressed as mean ± SEM. ns, $p > 0.05$.

f. Representative blots of CO-IP and quantification of the protein changes of NLRP3 in two groups (n = 3 mouse tissue samples). Student's *t*-test (P = 0.4035). Data are expressed as mean ± SEM. ns, $p > 0.05$.

g. Representative TEM images of ER-mitochondria contact and the quantitative analysis of the distance between mitochondria and the ER (n = 3 mitochondria in 3 fields per condition). Scale bar: 2 μm. Student's *t*-test (P = 0.7304). Data are expressed as mean ± SEM. ns, $p > 0.05$. Source data are provided as a Source Data file.

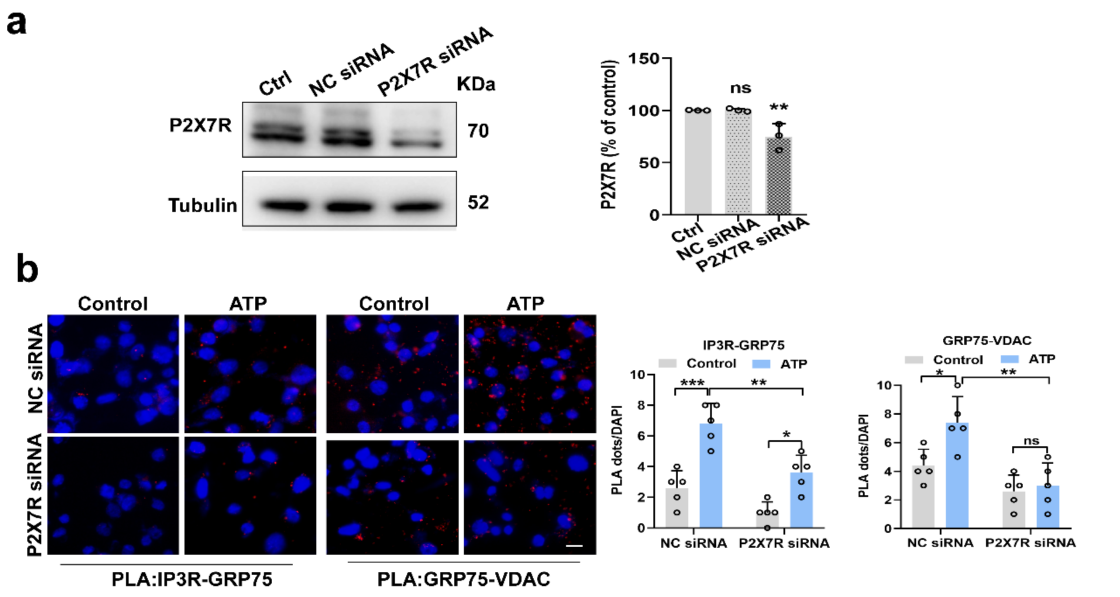

Supplementary Figure 5. Related to Figure 5: P2X7 Receptors mediate eATP-induced alterations in MAMs.

a. Western blot analysis of P2X7R proteins from BV2 cells subjected to transfection of P2X7R siRNA or negative control (NC) siRNA. One-way ANOVA with Dunnett's multiple comparisons test (P = 0.9994; P = 0.0098). Data are expressed as mean ± SEM (n = 3). ns, $p > 0.05$, $^{**}p < 0.01$, *vs.* Ctrl.

b. Representative images of proximity ligation assay (PLA) targeting IP3R-GRP75 or GRP75-VDAC interactions (left panel) and quantification of the PLA red fluorescent dots (right panel) in BV2 cells after transfected with P2X7R or NC siRNA. Scale bar: 20 μm; Two-way ANOVA with Sidak's multiple comparisons test (Interaction, $F_{(1, 16)} = 2.667$, P = 0.1220; Interaction, $F_{(1, 16)} = 4.024$, P = 0.0621). Data are expressed as mean ± SEM (n = 5). ns, $p > 0.05$, $^{*}p < 0.05$, $^{**}p < 0.01$. Source data are provided as a Source Data file.

Supplementary Figure 6. Example of original western blot for three or four repeats related to figure3.

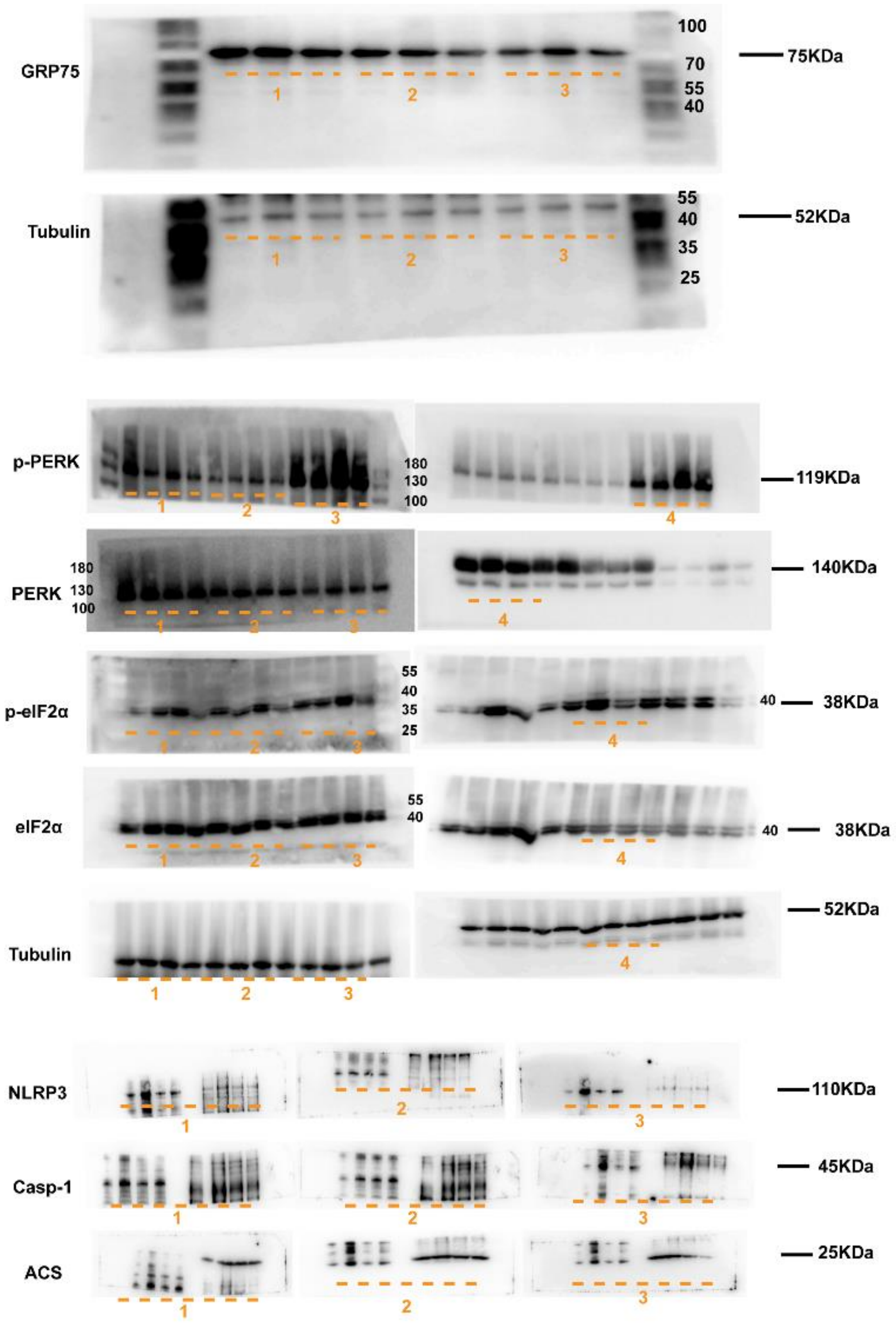

Supplementary Figure 7. Example of original western blot for three repeats related to figure4.

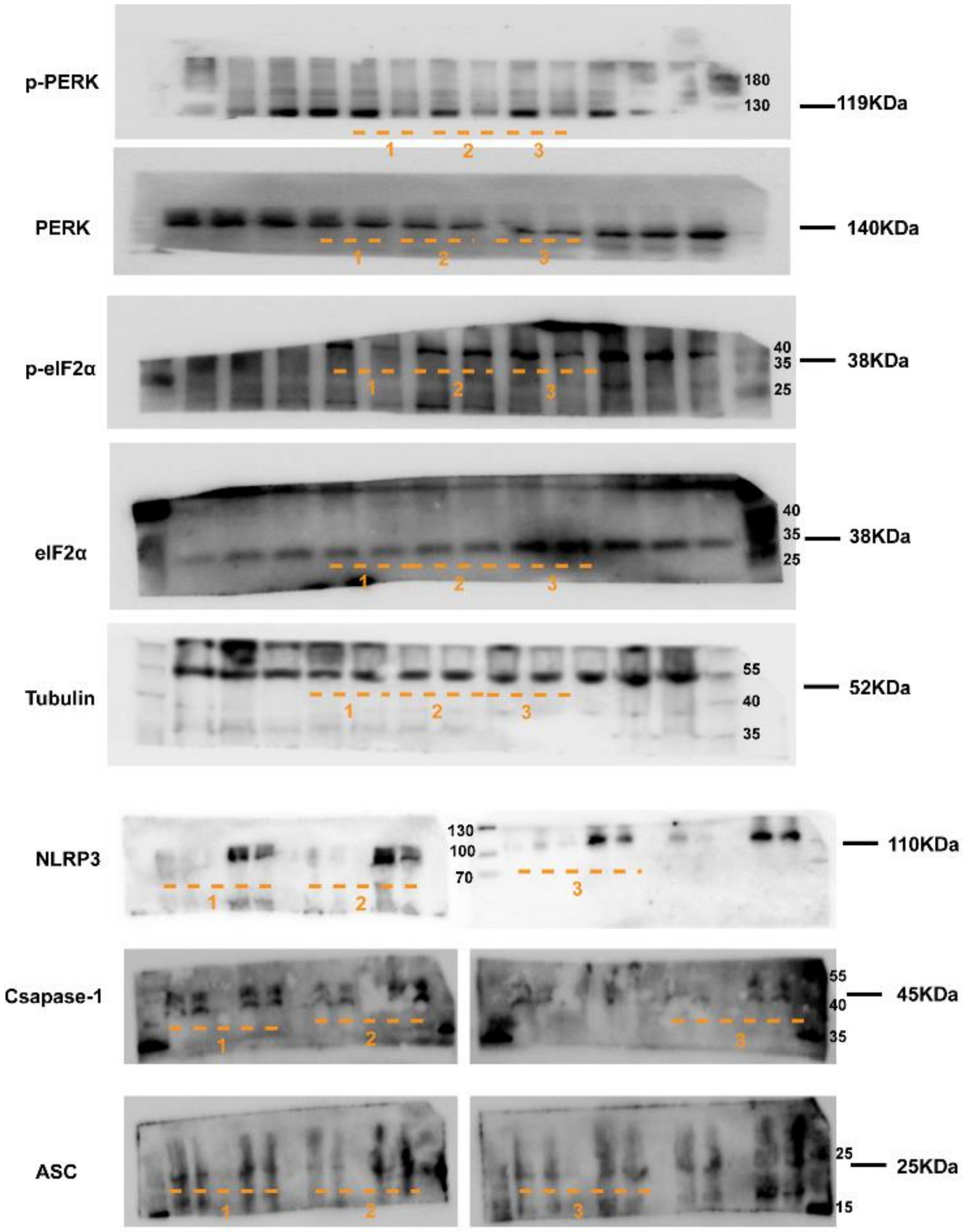

Supplementary Figure 8. Example of original western blot for three repeats related to sfigure3.

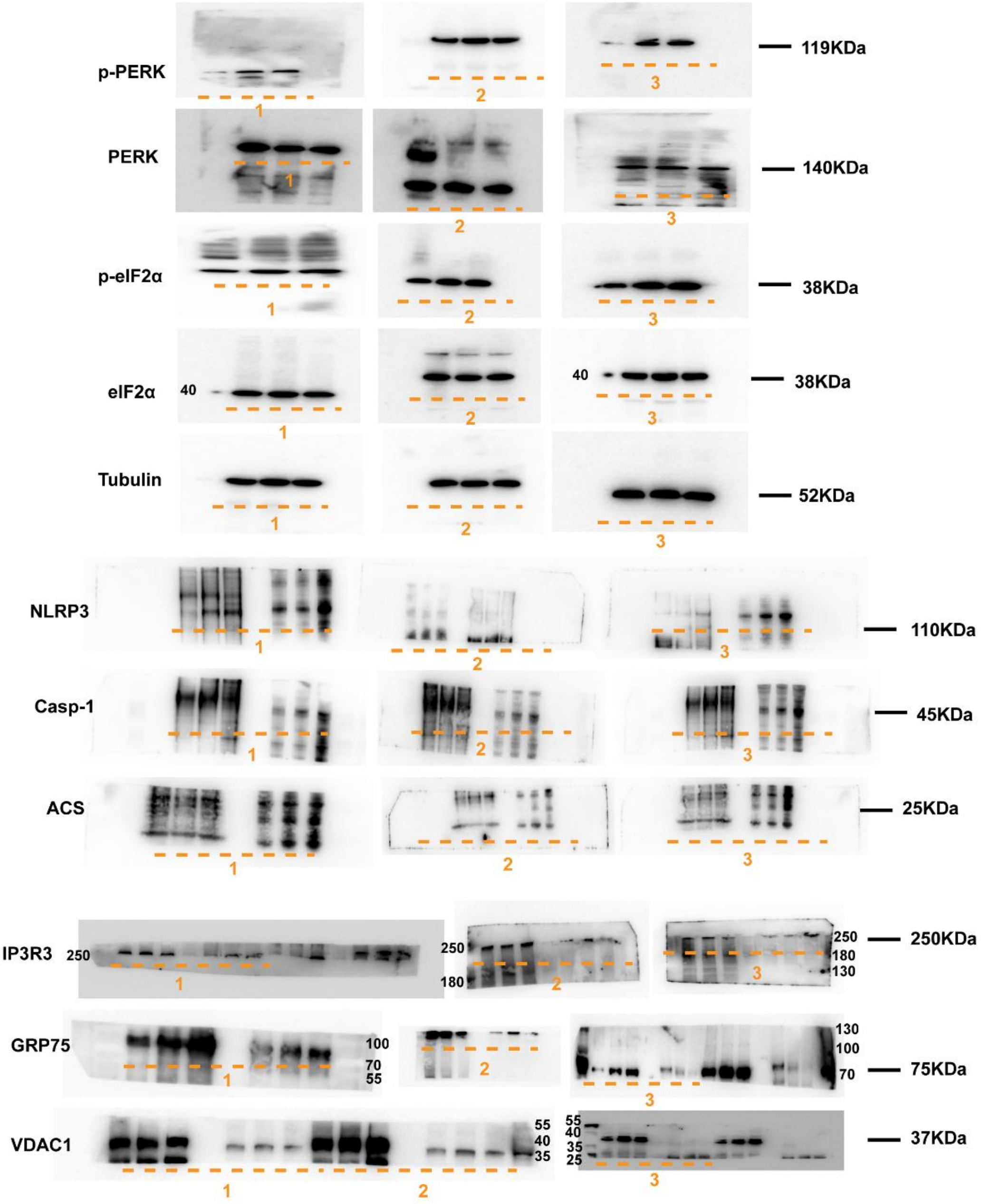

Supplementary Figure 9. Example of original western blot for three repeats related to sfigure4.

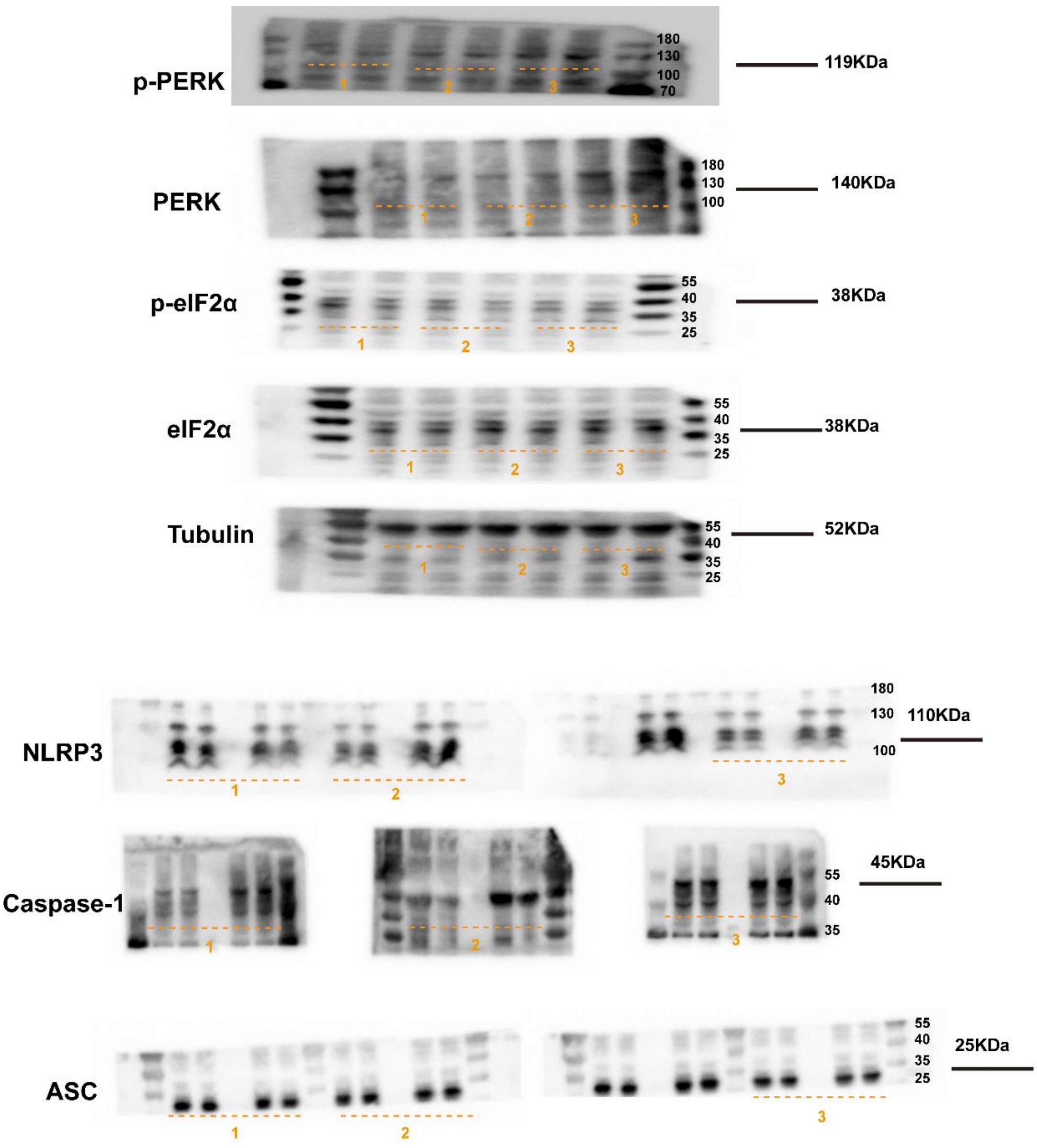

Supplementary Figure 10. Example of original western blot for three repeats related to sfigure5.

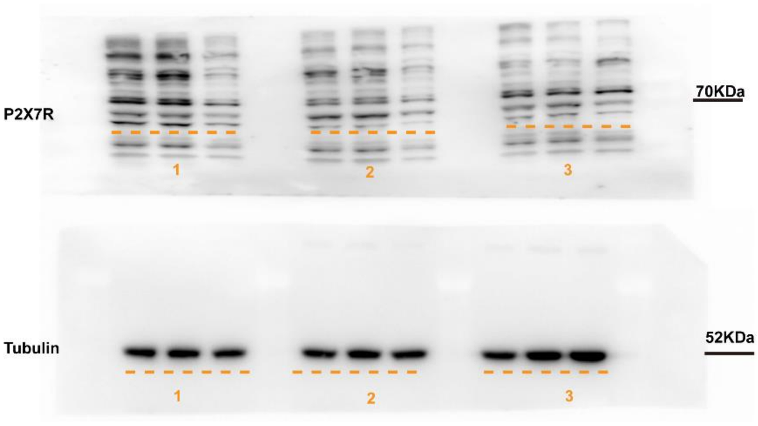

Supplementary Table 1 Related primers of target genes in quantitative PCR

Gene	Sense Primer (5'-3')	Antisense Primer (3'-5')
CD68	TCTGATCTTGCTAGGACCGCTTAT	GGCTGGCTGTGCTTTCTGTG
Cx3cr1	CACTGTTGCCTCAACCCCTTTA	ATGCTGTCCTGCCTGCTCCT
MCH II	AGGCAACTTTGGGCTGTGAG	CTTGTTCTGCTGGGTGGAGG
CD40	AGAAGACCCAATGCCACCCA	AGCACATGCCTCGCAATCC
Arg1	CTGCATATCTGCCAAAGACATCG	CCATCACCTTGCCAATCCC
GAPDH	AAGAAGGTGGTGAAGCAGG	GAAGGTGGAAGAGTGGGAGT
