# Supplementary material for: Augmented microglial endoplasmic reticulum-mitochondria contacts mediate depression-like behavior in mice induced by chronic social defeat stress
Source: Nat Commun. 2024 Jun 18;15:5199. doi: 10.1038/s41467-024-49597-z (PMC11189428; doi:10.1038/s41467-024-49597-z)
Supplement: Supplementary file 3 — Reporting Summary [file 41467_2024_49597_MOESM3_ESM.pdf]

nature portfolio

Corresponding author(s): Jin Yu

Last updated by author(s): Jun 2, 2024

Reporting Summary

Nature Portfolio wishes to improve the reproducibility of the work that we publish. This form provides structure for consistency and transparency in reporting. For further information on Nature Portfolio policies, see our Editorial Policies and the Editorial Policy Checklist.

Statistics

For all statistical analyses, confirm that the following items are present in the figure legend, table legend, main text, or Methods section.

n/a	Confirmed	
[ ]	[x]	The exact sample size (*n*) for each experimental group/condition, given as a discrete number and unit of measurement
[ ]	[x]	A statement on whether measurements were taken from distinct samples or whether the same sample was measured repeatedly
[ ]	[x]	The statistical test(s) used AND whether they are one- or two-sided<br>*Only common tests should be described solely by name; describe more complex techniques in the Methods section.*
[x]	[ ]	A description of all covariates tested
[x]	[ ]	A description of any assumptions or corrections, such as tests of normality and adjustment for multiple comparisons
[ ]	[x]	A full description of the statistical parameters including central tendency (e.g. means) or other basic estimates (e.g. regression coefficient) AND variation (e.g. standard deviation) or associated estimates of uncertainty (e.g. confidence intervals)
[ ]	[x]	For null hypothesis testing, the test statistic (e.g. *F*, *t*, *r*) with confidence intervals, effect sizes, degrees of freedom and *P* value noted<br>*Give P values as exact values whenever suitable.*
[x]	[ ]	For Bayesian analysis, information on the choice of priors and Markov chain Monte Carlo settings
[x]	[ ]	For hierarchical and complex designs, identification of the appropriate level for tests and full reporting of outcomes
[ ]	[x]	Estimates of effect sizes (e.g. Cohen's *d*, Pearson's *r*), indicating how they were calculated

Our web collection on statistics for biologists contains articles on many of the points above.

Software and code

Policy information about availability of computer code

Data collection	Behavioral test data were collected using small animal behavior analysis system. Flow cytometry data were collected using the BD, FACSVerse. Quantitative PCR data were obtained with ThermoFisher 7300Plus Real-Time PCR System. Immunoblotting images were obtained using the GE ImageQuantLAS4000 Chemiluminescence imaging system.
Data analysis	Behavioral test data were analysed using video tracking software (Ethovision 5.0, Noldus).Flow cytometry data were analysed using Flow Jo Software (Treestar, FlowJo 10.6.2). Immunofluorescent images were analyzed using ImageJ (v1.8.0). Statistical analysis was performed using Graphpad Prism (GraphPad software, version 8). Figure picture assembled using Adobe Photoshop CC 2018 (v19.1.3)

For manuscripts utilizing custom algorithms or software that are central to the research but not yet described in published literature, software must be made available to editors and reviewers. We strongly encourage code deposition in a community repository (e.g. GitHub). See the Nature Portfolio guidelines for submitting code & software for further information.

Data

Policy information about availability of data

All manuscripts must include a data availability statement. This statement should provide the following information, where applicable:

- Accession codes, unique identifiers, or web links for publicly available datasets
- A description of any restrictions on data availability
- For clinical datasets or third party data, please ensure that the statement adheres to our policy

Data Availability Statement. Bulk RNA sequencing data that support the findings of this study has been deposited in NCBI BioProject with the primary accession code

PRJNA1119092 (https://www.ncbi.nlm.nih.gov/bioproject/1119092) and in SRA with the primary accession codes SRX24775092, SRX24775093, SRX24775094, SRX24775095, SRX24775096 and SRX24775097 (https://www.ncbi.nlm.nih.gov/sra/PRJNA1119092). Source data are provided as a Source Data file. All other data is available from the corresponding author upon request.

Research involving human participants, their data, or biological material

Policy information about studies with human participants or human data. See also policy information about sex, gender (identity/presentation), and sexual orientation and race, ethnicity and racism.

Reporting on sex and gender

Use the terms sex (biological attribute) and gender (shaped by social and cultural circumstances) carefully in order to avoid confusing both terms. Indicate if findings apply to only one sex or gender; describe whether sex and gender were considered in study design; whether sex and/or gender was determined based on self-reporting or assigned and methods used.
Provide in the source data disaggregated sex and gender data, where this information has been collected, and if consent has been obtained for sharing of individual-level data; provide overall numbers in this Reporting Summary. Please state if this information has not been collected.
Report sex- and gender-based analyses where performed, justify reasons for lack of sex- and gender-based analysis.

Reporting on race, ethnicity, or other socially relevant groupings

Please specify the socially constructed or socially relevant categorization variable(s) used in your manuscript and explain why they were used. Please note that such variables should not be used as proxies for other socially constructed/relevant variables (for example, race or ethnicity should not be used as a proxy for socioeconomic status).
Provide clear definitions of the relevant terms used, how they were provided (by the participants/respondents, the researchers, or third parties), and the method(s) used to classify people into the different categories (e.g. self-report, census or administrative data, social media data, etc.)
Please provide details about how you controlled for confounding variables in your analyses.

Population characteristics

Describe the covariate-relevant population characteristics of the human research participants (e.g. age, genotypic information, past and current diagnosis and treatment categories). If you filled out the behavioural & social sciences study design questions and have nothing to add here, write "See above."

Recruitment

Describe how participants were recruited. Outline any potential self-selection bias or other biases that may be present and how these are likely to impact results.

Ethics oversight

Identify the organization(s) that approved the study protocol.

Note that full information on the approval of the study protocol must also be provided in the manuscript.

Field-specific reporting

Please select the one below that is the best fit for your research. If you are not sure, read the appropriate sections before making your selection.

- [x] Life sciences
- [ ] Behavioural & social sciences
- [ ] Ecological, evolutionary & environmental sciences

For a reference copy of the document with all sections, see nature.com/documents/nr-reporting-summary-flat.pdf

Life sciences study design

All studies must disclose on these points even when the disclosure is negative.

Sample size

For in vivo studies, at least 6 animals were checked for behavioral testing and at least 3 animals for molecular experiments. For in vitro studies, at least 3 samples per group to detect meaningful biological differences.

Data exclusions

No data or animals were excluded from analysis

Replication

Except for the animal studies (one time), RNA-seq analysis (one time), each experiment was repeated at least three times with similar results.

Randomization

Mice were randomly assigned to different groups.

Blinding

Blinding was not performed both in vivo or in vitro experiments, because the investigator needed to know the treatment groups in order to perform the study.

Reporting for specific materials, systems and methods

We require information from authors about some types of materials, experimental systems and methods used in many studies. Here, indicate whether each material, system or method listed is relevant to your study. If you are not sure if a list item applies to your research, read the appropriate section before selecting a response.

Materials & experimental systems

n/a	Involved in the study
[ ]	[x] Antibodies
[ ]	[x] Eukaryotic cell lines
[x]	[ ] Palaeontology and archaeology
[ ]	[x] Animals and other organisms
[x]	[ ] Clinical data
[x]	[ ] Dual use research of concern
[x]	[ ] Plants

Methods

n/a	Involved in the study
[x]	[ ] ChIP-seq
[ ]	[x] Flow cytometry
[x]	[ ] MRI-based neuroimaging

Antibodies

Antibodies used	1. Mouse anti-IP3R3 Santa Cruz Biotechnology sc-377518 1/200 dilution<br>2. Rabbit anti-p-PERK Bioss Antibodies bs-23340R 1/1000 dilution<br>3. Rabbit anti-PERK Cell Signaling technology 5683 1/1000 dilution<br>4. Rabbit anti-P-elF2α Cell Signaling technology 3597 1/1000 dilution<br>5. Rabbit anti-elF2α Cell Signaling technology 9722 1/1000 dilution<br>6. Rabbit anti-NLRP3 Proteintech 19771-1-AP 1/1000 dilution<br>7. Rabbit anti-ASC Proteintech 10500-1-AP 1/1000 dilution<br>8. Rabbit anti-Caspase1 Proteintech 22915-1-AP 1/1000 dilution<br>9. Mouse anti-VDAC Abcam ab14734, RRID:AB_443084 1/2000 dilution<br>10. Rabbit anti-Iba1/AIF-1 Cell Signaling technology 17198; RRID:AB_2820254 1/500 dilution<br>11. Mouse anti-P2X7R Santa Cruz Biotechnology sc-514962 1/500 dilution<br>12. Rabbit anti-GRP75 Proteintech 14887-1-AP 1/1000 dilution<br>13. Rabbit anti-α-tubulin Cell Signaling technology 5335 1/5000 dilution<br>14. Rabbit anti-GAPDH Cell Signaling technology 5174 1/5000 dilution<br>15. Goat anti-Mouse HRP Bio-Rad 1721011, RRID:AB_2617113 1/1000 dilution<br>16. Goat anti-Rabbit HRP Bio-Rad 1706515, RRID:AB_11125142 1/1000 dilution<br>17. Mouse IgG Beyotime A7028 1/1000 dilution<br>18. Rabbit IgG Beyotime A7016 1/1000 dilution<br>19.Goat anti-Rabbit IgG H&L (Alexa Fluor 488) Abcam ab150077 1/500 dilution<br>20.Goat anti-Mouse IgG H&L (Alexa Fluor 594) Abcam ab150116 1/500 dilution
Validation	All antibodies used are commercially available and validated by the manufacturers. The validation information in manufacture website.

Eukaryotic cell lines

Policy information about cell lines and Sex and Gender in Research

Cell line source(s)	The BV2 murine microglial cell line was acquired from the Cell Bank of the Chinese Academy of Sciences (Shanghai, China)
Authentication	N/A
Mycoplasma contamination	No
Commonly misidentified lines (See ICLAC register)	No commonly misidentified cell lines were used.

Animals and other research organisms

Policy information about studies involving animals; ARRIVE guidelines recommended for reporting animal research, and Sex and Gender in Research

Laboratory animals	Eight-week-old C57BL/6J mice and seven-month-old CD1 mice were obtained from Slack Laboratory Animal Co., Ltd. (Shanghai, China), P2X7R-/- mice were purchased from the Shanghai Experimental Animal Center of the Chinese Academy of Sciences and the Jackson Laboratory (Shanghai, China, Cat. 005576). Conditional microglia-specific Hspa9 knockout mice were generated using the Cre/LoxP system. Hspa9flox/+ mice (Strain NO. T008726) under C57BL/6J background were purchased from GemPharmatech LLC. (Nanjing, China). Cx3cr1CreER mice (Strain #:021160) were purchased from The Jackson Laboratory (Maine, U.S.A). All animals were habituated in 12 h light/dark cycle and allowed free access to food and water under conditions of controlled humidity and temperature (24 ± 0.5 °C).
Wild animals	This study did not involve wild animals.
Reporting on sex	Male mice were used in the experiments.

Field-collected samples	This study did not involve samples collected in the field.
Ethics oversight	All experiments were carried out in accordance with the National Institutes of Health Guide for the Care and Use of Laboratory Animals and approved by the Experimental Animal Ethics Committee of Shanghai Medical College, Fudan University, Shanghai, China (20160225-071).

Note that full information on the approval of the study protocol must also be provided in the manuscript.

Plants

Seed stocks	*Report on the source of all seed stocks or other plant material used. If applicable, state the seed stock centre and catalogue number. If plant specimens were collected from the field, describe the collection location, date and sampling procedures.*
Novel plant genotypes	*Describe the methods by which all novel plant genotypes were produced. This includes those generated by transgenic approaches, gene editing, chemical/radiation-based mutagenesis and hybridization. For transgenic lines, describe the transformation method, the number of independent lines analyzed and the generation upon which experiments were performed. For gene-edited lines, describe the editor used, the endogenous sequence targeted for editing, the targeting guide RNA sequence (if applicable) and how the editor was applied.*
Authentication	*Describe any authentication procedures for each seed stock used or novel genotype generated. Describe any experiments used to assess the effect of a mutation and, where applicable, how potential secondary effects (e.g. second site T-DNA insertions, mosiacism, off-target gene editing) were examined.*

Flow Cytometry

Plots

Confirm that:

☒ The axis labels state the marker and fluorochrome used (e.g. CD4-FITC).

☒ The axis scales are clearly visible. Include numbers along axes only for bottom left plot of group (a 'group' is an analysis of identical markers).

☒ All plots are contour plots with outliers or pseudocolor plots.

☒ A numerical value for number of cells or percentage (with statistics) is provided.

Methodology

Sample preparation	Cells were pretreated with TG or ATP, JC-1 working solution was added to the culture medium and incubated at 37 centigrade for 30 min. After washing twice with cold dyeing buffer and the subsequent centrifugation, the cells were resuspended in the dyeing buffer.
Instrument	Cells were analyzed by flow cytometry (BD, FACSVerse, New York, USA).
Software	The data were analyzed using Flow Jo Software (Treestar, FlowJo 10.6.2).
Cell population abundance	At least 10,000 cells were analyzed for each sample.
Gating strategy	Gating strategies are described in the Methods section (Mitochondrial transmembrane potential (ΔΨm) assay)

☐ Tick this box to confirm that a figure exemplifying the gating strategy is provided in the Supplementary Information.
